# Psychometric assessment of three newly developed implementation outcome measures

**DOI:** 10.1186/s13012-017-0635-3

**Published:** 2017-08-29

**Authors:** Bryan J. Weiner, Cara C. Lewis, Cameo Stanick, Byron J. Powell, Caitlin N. Dorsey, Alecia S. Clary, Marcella H. Boynton, Heather Halko

**Affiliations:** 1 Department of Global Health, University of Washington, 1510 San Juan Road, Box 357965, Seattle, WA 98195 USA; 2 Kaiser Permanente Washington Health Research Institute, MacColl Center for Health Care Innovation, 1730 Minor Avenue, Suite 1600, Seattle, WA 98101 USA; 3 Department of Psychological and Brain Sciences, Indiana University, 1101 E 10th Street, Bloomington, IN 47405 USA; 4 Department of Psychiatry and Behavioral Sciences, University of Washington, 325 Ninth Street, Seattle, WA 98104 USA; 5 Hathaway-Sycamores Child and Family Services, 210 S DeLacey Ave, Suite 110, Pasadena, CA 91105-2074 USA; 6 Department of Health Policy and Management, University of North Carolina at Chapel Hill, 135 Dauer Drive, Chapel Hill, NC 27599 USA; 7 Department of Health Behavior, University of North Carolina at Chapel Hill, 135 Dauer Drive, Chapel Hill, NC 27599 USA; 8 Department of Psychology, University of Montana, Missoula, USA

**Keywords:** Acceptability of Intervention Measure (AIM), Intervention Appropriateness Measure (IAM), Feasibility of Intervention Measure (FIM), Implementation research, Acceptability, Feasibility, Appropriateness, Implementation outcomes, Measure, Structural validity, Test-retest, Known-groups

## Abstract

**Background:**

Implementation outcome measures are essential for monitoring and evaluating the success of implementation efforts. Yet, currently available measures lack conceptual clarity and have largely unknown reliability and validity. This study developed and psychometrically assessed three new measures: the Acceptability of Intervention Measure (AIM), Intervention Appropriateness Measure (IAM), and Feasibility of Intervention Measure (FIM).

**Methods:**

Thirty-six implementation scientists and 27 mental health professionals assigned 31 items to the constructs and rated their confidence in their assignments. The Wilcoxon one-sample signed rank test was used to assess substantive and discriminant content validity. Exploratory and confirmatory factor analysis (EFA and CFA) and Cronbach alphas were used to assess the validity of the conceptual model. Three hundred twenty-six mental health counselors read one of six randomly assigned vignettes depicting a therapist contemplating adopting an evidence-based practice (EBP). Participants used 15 items to rate the therapist’s perceptions of the acceptability, appropriateness, and feasibility of adopting the EBP. CFA and Cronbach alphas were used to refine the scales, assess structural validity, and assess reliability. Analysis of variance (ANOVA) was used to assess known-groups validity. Finally, half of the counselors were randomly assigned to receive the same vignette and the other half the opposite vignette; and all were asked to re-rate acceptability, appropriateness, and feasibility. Pearson correlation coefficients were used to assess test-retest reliability and linear regression to assess sensitivity to change.

**Results:**

All but five items exhibited substantive and discriminant content validity. A trimmed CFA with five items per construct exhibited acceptable model fit (CFI = 0.98, RMSEA = 0.08) and high factor loadings (0.79 to 0.94). The alphas for 5-item scales were between 0.87 and 0.89. Scale refinement based on measure-specific CFAs and Cronbach alphas using vignette data produced 4-item scales (α’s from 0.85 to 0.91). A three-factor CFA exhibited acceptable fit (CFI = 0.96, RMSEA = 0.08) and high factor loadings (0.75 to 0.89), indicating structural validity. ANOVA showed significant main effects, indicating known-groups validity. Test-retest reliability coefficients ranged from 0.73 to 0.88. Regression analysis indicated each measure was sensitive to change in both directions.

**Conclusions:**

The AIM, IAM, and FIM demonstrate promising psychometric properties. Predictive validity assessment is planned.

**Electronic supplementary material:**

The online version of this article (doi:10.1186/s13012-017-0635-3) contains supplementary material, which is available to authorized users.

## Background

Implementation outcomes play a critical role in implementation research and practice. Acceptability, appropriateness, feasibility, adoption, fidelity, cost, penetration, and sustainability serve not only as indicators of the effects of implementation processes (i.e., outcomes in their own right) but also as preconditions for attaining desired service delivery and clinical outcomes (i.e., intermediate outcomes) [1]. Reliable, valid, and pragmatic measures of these outcomes are essential for monitoring and evaluating the success of implementation efforts and comparing the effectiveness of alternative implementation strategies.

Despite their importance, existing measures of implementation outcomes have largely unknown psychometric and pragmatic qualities [2]. This status quo raises questions about these measures’ utility for building cumulative knowledge in implementation research or guiding implementation efforts in clinical and community settings. For example, most available measures have not been empirically tested for substantive or discriminant content validity. They are rarely defined or distinguished from one another and often employ similar items to assess purportedly different constructs [1, 3]. As a result, it is not clear exactly what these measures are assessing. Moreover, although most implementation outcome measures have been assessed for scale reliability, typically in the form of inter-item consistency, few if any have been assessed for other measurement properties that are of key importance to implementation researchers and other implementation stakeholders (e.g., intermediaries, policymakers, practice leaders), such as stability, responsiveness, and predictive validity [2–4]. Importantly, existing measures of implementation outcomes have not been administered together in research studies, leaving open the question of whether some of these outcomes, which are conceptually distinguishable, are empirically distinguishable [1].

In this article, we report the results of a psychometric assessment of newly developed measures of three implementation outcomes: acceptability (Acceptability of Intervention Measure (AIM)), appropriateness (Intervention Appropriateness Measure (IAM)), and feasibility (Feasibility of Intervention Measure (FIM)). We selected these implementation outcomes because (a) they are often used in formative research or pilot studies as “leading indicators” of implementation success [1, 5] and (b) they are conceptually distinct but likely to be empirically inter-related in complex ways [1]. Evidence that two or more of these outcomes are highly correlated with each other would suggest that they could serve as proxies for each other. We created new measures of these outcomes because our preliminary work suggested that addressing the definitional ambiguities and overlapping item content in existing measures would have entailed substantial adaptations such that new measures would essentially be created. Consistent with the overarching goal of our team’s work to enhance the psychometric strength and pragmatic quality of measures in implementation science [4], we involved researchers and other stakeholders in the measure development and testing process.

## Conceptual framework

Proctor and her colleagues defined acceptability, appropriateness, and feasibility as follows [1]:

*Acceptability* is the perception among implementation stakeholders that a given treatment, service, practice, or innovation is agreeable, palatable, or satisfactory…. *Appropriateness* is the perceived fit, relevance, or compatibility of the innovation or evidence-based practice for a given practice setting, provider, or consumer; and/or perceived fit of the innovation to address a particular issue or problem.…*Feasibility* is defined as the extent to which a new treatment, or an innovation, can be successfully used or carried out within a given agency or setting.

All three constructs imply an evaluation of the fit or match of something (e.g., an evidence-based practice (EBP)) and some criterion. For acceptability, the criterion is personal. Two people can view the same EBP and form different judgments about its acceptability to them if their needs, preferences, or expectations differ. For appropriateness, the criterion is technical or social. An EBP can be judged appropriate if it is seen as efficacious for achieving some purpose given existing conditions, including patients’ presenting problems, or seen as consistent with norms or values governing people’s conduct in particular situations, including organizational mission and treatment philosophy. For feasibility, the criterion is practical. An EBP can be judged feasible if a task or an action can be performed relatively easily or conveniently given existing resources (e.g., effort, time, and money) and circumstances (e.g., timing or sociopolitical will). These three constructs are semantically similar, as evidenced by some overlap in the synonyms for each in the dictionary; however, they can be distinguished conceptually and operationally based on the criterion that each references in the evaluation of fit or match.

These three constructs could be difficult to distinguish empirically if an antecedent condition meets more than one criterion upon which fit or match is evaluated. For example, when mandated, as EBP could be judged both as unacceptable if a provider regards its required use as undercutting her clinical autonomy and as inappropriate if a provider sees it as contrary to organizational treatment philosophies. Similarly, when standardized, an EBP could be judged both as inappropriate, if a provider regards it as ineffective in meeting patients’ varied needs, and as infeasible if a provider sees it as difficult to implement given her organization’s staffing and resources. Although perceptions of EBP acceptability, appropriateness, and feasibility are likely to covary frequently, it is possible to imagine, create, or observe conditions in which they do not. For example, given the criteria for evaluating fit or match described above and drawing on constructs in the Consolidated Framework for Implementation Research [6], we expect that acceptability is likely to vary to a greater extent than either appropriateness or feasibility as a function of individuals’ willingness to try new things (openness) on a limited, revocable basis (trialability). Appropriateness is likely to vary to a greater extent than either acceptability or feasibility as a function of consistency with professional values (norms) or perceived efficacy in meeting patient needs (relevance). Feasibility is likely to vary to a greater extent than either acceptability or appropriateness as a function of cost or time (resource availability) or ease of implementation or use (complexity). Proctor and colleagues propose that these three implementation outcomes are most salient in the adoption phase of the innovation-decision process. Although predictive validity is not assessed below, acceptability, appropriateness, and feasibility are hypothesized to be associated with EBP adoption [1].

## Methods

We conducted three studies to assess the psychometric properties of the newly developed measures of acceptability (AIM), appropriateness (IAM), and feasibility (FIM). In study 1, we assessed the measures’ substantive and discriminant content validity by administering a web-based survey to a sample of implementation researchers and implementation-experienced mental health professionals. In study 2, we examined the structural validity, reliability, and known-groups validity of the three constructs by conducting an experimental vignette study with mental health counselors. In study 3, we examined the measures’ test-retest reliability and sensitivity to change by re-administering the experiment to the same participants several weeks after study 2.

## Study 1

### Method

Substantive validity refers to the extent to which a measure is judged to be reflective of, or theoretically linked to, some construct of interest [7]. When substantive validity is demonstrated for several measures simultaneously, discriminant content validity is also demonstrated [8]. Typically, substantive validity is assessed informally and qualitatively by asking a few researchers (usually colleagues of the measure developer) to consider whether a measure’s items seem representative of the construct’s theoretical content [9]. In this study, we took a formal, quantitative approach by asking a large group of researchers and practitioners to rate the extent to which items reflect the constructs they were intended to measure [8].

#### Design, participants, and procedures

Fifty-one Ph.D.-trained implementation scientists and 52 implementation-experienced mental health professionals were recruited by email through the Society for Implementation Research Collaboration (SIRC) [10] and the authors professional networks. Thirty-six implementation scientists and 27 mental health professionals agreed to participate in the study. By involving both researchers and practitioners, we sought to increase the relevance of the measures to both stakeholder groups [11]. Sixty-eight percent of the study participants identified as male, 94% identified as Caucasian, 3% identified as African American, and 3% identified as Asian. Eleven percent identified as Hispanic. Seventy-eight percent of the implementation scientists reported receiving funding as a principal investigator to study or evaluate implementation. Seventeen percent of the mental health professionals were administrators; 13% were clinicians. One professional identified as both. On average, these professionals had 8 years of experience implementing EBPs or leading EBP implementation projects in mental health settings.

The study participants completed a web-based survey in which the three constructs were defined at the top of the survey and 31 items reflecting the three constructs were listed below in random order. The participants assigned each item to the construct that they perceived the item measured. The participants could assign an item to more than one construct. They rated their confidence in each assignment from 0% for “not at all confident” to 100% for “extremely confident.” Correct assignments (i.e., items assigned to intended constructs) were coded 1 (i.e., a “match”). Incorrect assignments were coded − 1 (i.e., “no match”). The survey design ensured that no missing data occurred for assignments. Each assignment was multiplied by its accompanying confidence rating. Thus, weighted assignments ranged from − 1 to 1.

#### Measures

We employed a deductive approach to item generation [9], whereby we used the definitions and conceptual framework described above to ascertain whether items from existing measures, obtained through the Society for Implementation Research Collaboration [12] and literature searches, adequately capture the theoretical content of the construct. Based on our findings, 12 items were generated to measure acceptability (e.g., “This EBP seems fine”), 11 items were written to measure appropriateness (e.g., “This EBP seems suitable.”), and 8 items were produced to measure feasibility (e.g., “This EBP seems doable.”). See Table 1. The appropriateness items did not specify a purpose (e.g., “for treating depression”), person (e.g., “for my patients”), or situation (e.g., “for my organization”) in order to keep the substantive validity assessment of these items general rather than restricted.

**Table 1:** Discriminant content validity analysis, *N* = 63

Construct	Item	Median	Wilx	
Acceptability	This EBP seems fine.	.1	.012	
	This EBP seems good enough.	0	.163	ns
	This EBP will do.	.1	.062	ns
	This EBP meets my approval.	.8	.000	
	This EBP meets my needs.	.2	.006	
	This EBP is okay.	.2	.005	
	This EBP is satisfactory.	.6	.000	
	I have no objection to this EBP.	.7	.000	
	This EBP is pretty good.	0	.027	ns
	This EBP is appealing.	.7	.000	
	I like this EBP.	.8	.000	
	I welcome use of this EBP	.8	.000	
Appropriateness	This EBP seems right.	0	.174	ns
	This EBP seems fitting.	.75	.000	
	This EBP seems suitable.	.7	.000	
	This EBP seems reasonable.	− .5	.000	
	This EBP seems applicable.	.75	.000	
	This EBP seems right on the button.	.2	.050	ns
	This EBP seems proper.	.6	.000	
	This EBP seems apt.	.7	.000	
	This EBP seems like a good match.	.8	.000	
	This EBP seems well aligned.	.6	.000	
Feasibility	This EBP seems practical.	.25	.002	
	This EBP seems realistic.	.6	.000	
	This EBP seems workable.	.7	.000	
	This EBP seems implementable.	.9	.000	
	This EBP seems possible.	.9	.000	
	This EBP seems viable.	.35	.000	
	This EBP seems doable.	1	.000	
	This EBP seems challenging.	.7	.000	
	This EBP seems easy to use.	.9	.000	

*ns* not significant at .05 level after false discovery rate controlling procedure for multiple tests

#### Data analysis

To assess substantive and discriminant content validity, we used the Wilcoxon one-sample signed rank test to determine whether items represented the intended construct more so than the other constructs (i.e., demonstrated discriminant content validity) [8]. Specifically, an item was said to measure a construct if its median weighted assignment to that construct was significantly greater than zero. The Wilcoxon test was used instead of the *t* test since the weighted assignments were not normally distributed. Hochberg’s correction was used to correct for multiple tests [13]. We calculated intraclass correlation coefficients (ICCs) using two-way mixed effects model to assess the level of agreement in item assignments among all participants, and also within stakeholder groups, across all 31 items and for each construct [14]. Weighted assignments were used to calculate ICCs.

To assess the validity of the conceptual model, we performed both exploratory and confirmatory factor analysis (EFA and CFA) on the same data using M*plus* 7 [15]. A separate five-factor EFA with promax oblique rotation for each construct was estimated to allow for the possibility of additional factors beyond the three expected factors, resulting from possible item cross-loadings, and to trim the number of items. A three-factor CFA model using robust weighted least-squares estimation was then tested using M*plus* 7 by loading the five best-performing items on their respective constructs. We used the following guidelines for determining good model fit: comparative fit index (CFI) equal to or greater than 0.95 and root mean square error of approximation (RMSEA) equal to or less than 0.08 [16, 17]. Power analysis based on the RMSEA test of close fit estimated power at 84% with a sample size of 60 participants [18]. Since the weighted assignments were not normally distributed, prior to running the EFA and CFA, we collapsed the weighted assignments into intervals spanning 20 points each (0 = 0, 1–19% = 1, 20–39% = 2, 40–59% = 3, 60–79% = 4, 80–99% = 5, and 100% = 6) and used the re-scaled items as categorical variables. This approach was consistent with the expressed distributions and variability of the responses.

### Results

Judges exhibited a high degree of consistency in their item assignments. The ICC for all participants across all 31 items was 0.89 (95% CI 0.84–0.92). The ICC for all participants for each construct was as follows: 0.82 (95% CI 0.63–0.94) for acceptability, 0.94 (95% CI 0.86–0.98) for appropriateness, and 0.87 (95% CI 0.72–0.96) for feasibility. Weighted assignments for both stakeholder groups—Ph.D.-trained implementation scientists and implementation-experienced mental health professionals—were pooled in subsequent analysis since no significant group differences were observed.

Table 1 displays the results of the discriminant content validity analysis. Median weighted assignments for all but five items were significantly greater than zero after applying the Hochberg correction for multiple tests. This indicated that the participants judged the items to reflect to a significantly greater degree the constructs they were intended to measure than they did the other constructs. The five items that did not have sufficient discriminant content validity were “This EBP is good enough,” “This EBP will do,” and “This EBP is pretty good” for acceptability and “This EBP seems right” and “This EBP seems right on the button” for appropriateness. The negative median weighted assignment for “This EBP seems reasonable” indicates that the respondents had greater confidence in assigning this item to acceptability or feasibility than to appropriateness. Hence, this item also lacks discriminant content validity.

The five-factor EFA model indicated that all but two items loaded on a single factor (see Additional file 1). The item “This EBP is fine” cross-loaded on the acceptability factor and a fourth, undefined factor. The item “This EBP seems reasonable” did not load on the appropriateness factor. For each construct, the five items with the strongest factor scores and inter-item correlations were selected for testing in a CFA model. All but one of these items (“This EBP seems well aligned”) was among the top-five mean weighted assignments for its intended construct. Internal consistency of the five-item scales was good: acceptability (α = .89), appropriateness (α = .87), and feasibility (α = .89).

**Fig. 1:**
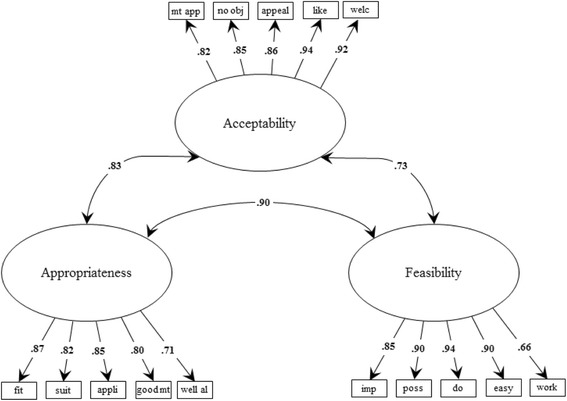
Confirmatory factor analysis of substantive validity data *N* = 63. χ^2^(87) = 120.6, *p* =.01; CFI = 0.98 ; RMSEA = 0.08, CI [0.04-0.11]. All path loadings are significant at *p* < .05

The CFA model exhibited factor loadings that ranged between .79 and .94 (see Fig. 1), and model fit was adequate, as evidenced by CFI = 0.98 and RMSEA = 0.08 (CI, 0.04–0.11). The correlations among the three factors were high, ranging from 0.77 to 0.91. Although these correlations are high, a CFA model loading all 15 items on a single factor exhibited poor model fit, as evidenced by CFI = 0.94 and RMSEA = 0.15 (CI, 0.13–0.18) and an increase in the BIC from 2493.5 for the three-factor model to 2565.4 for the one-factor model. These results suggested that the three-factor CFA model is a better representation of the factor structure of the scale items than a one-factor model.

## Study 2

### Method

In study 2, we ascertained the structural validity, reliability, and known-groups validity of our new measures in an online experimental study. Structural validity refers to the extent to which the inter-relationships among items measuring a construct accord with the expected internal structure of the construct [19]. Reliability here refers to the extent to which the items measuring a construct exhibit internal consistency [15]. Known-groups validity refers to the extent to which a measure is sensitive to known differences between groups [20].

#### Design, participants, and procedures

Study participants were a convenience sample of 326 counselors belonging to the American Mental Health Counselors Association (AMHCA). These participants were obtained through an email invitation sent to 2000 non-retired clinical members of AMHCA, with non-respondents receiving up to three reminder emails spaced a week apart. Twenty-eight percent of the 326 study participants identified as male, 71% as female, and one person as non-binary gender. Ninety-one percent identified as Caucasian, 5% as African American, 2% as Asian, and 2% as “Other.” Four percent identified as Hispanic, Latino, or Spanish origin. Seventy-seven percent indicated that they work in a private practice setting. Fifty-three percent reported that they had implemented an EBP or led the implementation of an EBP in the past 5 years.

The study participants read one of six randomly assigned vignettes in which a therapist discusses with a colleague her thoughts about adopting measurement-based care (MBC). MBC is an evidence-based practice that involves collecting client progress and outcome data on a routine basis using brief, validated measures and using those data to inform treatment decisions [21]. In each of the vignettes, the descriptive content of the therapist’s discussion was designed to systematically vary with respect to her assessment of MBC’s acceptability, appropriateness, and feasibility. Guided by the conceptual framework, we manipulated two levels (high versus low) of the three constructs by varying the following information in the vignettes: openness, trialability, relevance for patient needs, implementation complexity, and resource availability. The participants rated the therapist’s perceptions of the acceptability, appropriateness, and feasibility of adopting MBC based on the views that the therapist expressed in the vignette, not their own personal opinion of MBC. Due to a study design error, two of the eight possible vignettes were not fielded: one in which acceptability was high, appropriateness low, and feasibility high; and the other where acceptability was low, appropriateness high, and feasibility low. The six fielded vignettes manipulated all other combinations of the levels of the constructs. While unfortunate, this design error did not preclude us from assessing reliability, structural validity, or known-groups validity. However, it did limit our ability to explore discriminant validity, especially between acceptability and appropriateness, as two of the four possible vignettes in which these constructs were designed to move in opposite directions were not included. The vignettes were pilot-tested with a convenience sample of five Ph.D. implementation scientists and implementation-experienced mental health professionals and revised prior to use.

#### Measures

Using a 5-point ordinal scale that ranged from “completely disagree” to “completely agree,” participants rated the three constructs using the 15 items comprising the three 5-item scales developed in study 1. Item wording was changed slightly from study 1 to accommodate the hypothetical nature of the assessments. Specifically, the subject of each item was changed from first person to third person and from “EBP” to “MBC” (e.g., “I like this EBP” to “She likes MBC”). All items were randomly ordered within and across vignettes to avoid ordering effects.

#### Analysis

To refine the scales, we estimated separate CFAs for each scale, examined factor loadings for individual items, and computed Cronbach alphas for inter-item consistency. To assess structural validity, we estimated the same 3-factor CFA model as in study 1 using M*plus* 7 [15] using maximum likelihood estimation. For comparison purposes, we computed two other models: a 2-factor model with acceptability and appropriateness items loading on a single factor, and an omnibus 1-factor model -. We used the same CFA model fit guidelines as those employed in study 1. To assess known-groups validity, we conducted a 2^3^ analysis of variance (ANOVA) with the Tukey test for multiple comparisons, to determine whether the mean levels of the implementation outcomes (i.e., high versus low levels of acceptability, appropriateness, and feasibility) varied as expected based on information manipulation in the vignettes.

### Results

Factor loadings in the one-factor CFAs for each scale ranged from 0.84 to 0.92, with one exception (see Additional file 2). The item, “She has no objection to MBC,” exhibited a factor loading of 0.50. In order to trim the scales further, the poorest performing item in each of the scale was dropped. In the case of acceptability, the “no objection” item was dropped due to its low factor loading. The “MBC seems well aligned” and “MBC seems workable” items were dropped from the appropriateness and feasibility scales, respectively, based on their lower correlations with the other items in their respective scales. The Cronbach alphas for the trimmed 4-item scales were 0.85 for acceptability, 0.91 for appropriateness, and 0.89 for feasibility.

**Fig. 2:**
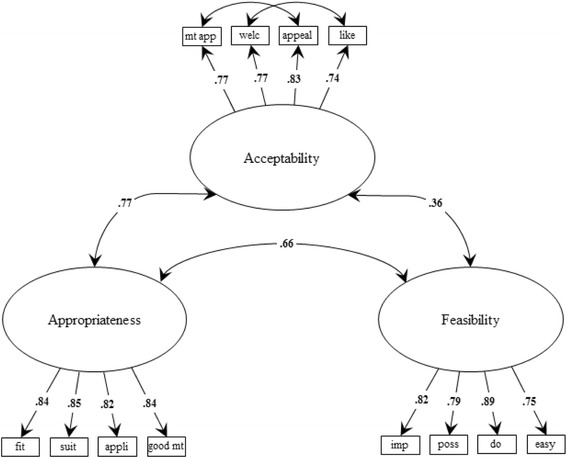
Confirmatory factor analysis of structural validity data *N* = 326. χ^2^(49) = 147.9, *p* = <.001; CFI = 0.96; RMSEA = 0.079, CI [0.06-0.09]. All path loadings are significant at *p* < .05

The factor loadings for the 3-factor CFA ranged from 0.75 to 0.89 and fit for the three-factor CFA model was acceptable. This was evidenced by CFI = 0.96 and RMSEA = 0.08 [CI, 0.06–0.09] [18, 22] after allowing the error terms between “MBC meets her approval” and “MBC is appealing to her” as well as between “She welcomes MBC” and “She likes MBC” to correlate to account for differences in sentence structure (i.e., passive versus active voice)(see Fig. 2). By comparison, the factor loadings for the omnibus 1-factor CFA model ranged from 0.55 to 0.84 and the model fit was quite poor (CFA = 0.73, RMSEA = .20 [CI, 0.19–0.21]). The 2-factor CFA model fit the data better than the one-factor model, with factor loading ranging from 0.63 to 0.89 and model fit improving (CFI = .89, RMSEA = .13 [CI, 0.12–0.14]). However, the 3-factor model fit the data better than either the 1-factor or 2-factor model. This was evidenced by the absolute model fit statistics referenced above as well as an increase in the BIC from 8499.4 for the 3-factor model to 8672.5 for the 2-factor model, with a further increase to 9085.5 for the 1-factor model. For the 2-factor model the correlations among the factors ranged from 0.36 to 0.77. The relatively high correlation between the acceptability and appropriateness scales (0.77) might have been inflated by the design error mentioned earlier. In four of the six vignettes fielded, these two constructs were designed to move in the same direction, either both high or both low. The correlation in these vignettes was 0.81. In the remaining two vignettes fielded, these constructs were designed to move in opposite directions, one high and the other low. The correlation in these vignettes was 0.36. Had the other two vignettes been fielded, in which these two constructs were also designed to move in opposite directions, the overall correlation of these two construct would likely have been lower than 0.77. In sum, these results indicate that the measures exhibit structural validity, although the discriminant validity of the acceptability and appropriateness measures is uncertain.

The 2^3^ ANOVAs revealed medium- to large-size main effects of each manipulation on the relevant scale score [23] (see Table 2). The manipulation of information designed to convey acceptability, appropriateness, and feasibility accounted for 42%, 37%, and 42% of the variance in the corresponding scale scores. The variance accounted for was reasonable given that study participants had to infer the level of acceptability, appropriateness, or feasibility in the vignettes. The medium-size main effects for acceptability in the appropriateness model and appropriateness in the acceptability model signals that respondents had some difficulty discriminating these two constructs. However, these constructs might have been overly conflated as a result of the loss of two vignettes in which these constructs were designed to move in opposite directions. The incomplete factorial design also precluded a full exploration of the two-way and three-way interactions. Nonetheless, the significant main effects provide preliminary evidence of known-groups validity: specifically, the three measures can differentiate groups with known (designed) differences in the levels of these constructs.

**Table 2:** ANOVA predicting acceptability, appropriateness, and feasibility of MBC, *N* = 326

		Dependent variables					
		Acceptability		Appropriateness		Feasibility	
Source	*df*	*F*	*η* ^2^	*F*	*η* ^2^	*F*	*η* ^2^
AC	1	114.1*	.26	20.3*	.06	4.1	.01
AP	1	15.4*	.05	66.0*	.17	4.9	.02
FE	1	0.58	.002	6.0*	.02	143.5*	.31
AC × AP	1	0.59	.002	0.18	.001	0.66	.00
AC × FE	1	0.48	.001	0.24	.001	1.3	.00
AP × FE							
AC × AP × FE							
Residual	320						
		*R* ^2^ = .43 (Adj *R* ^2^ = .42)		*R* ^2^ = .37 (Adj *R* ^2^ = .36)		*R* ^2^ = .42 (Adj *R* ^2^ = .41)	

The dependent variables are the acceptability, appropriateness, and feasibility 4-item scales. A design error precluded full exploration of two-way and three-way interactions

*AC* acceptability factor (high versus low, manipulated in vignette), *AP* appropriateness factor (high versus low, manipulated in vignette), *FE* feasibility factor (high versus low, manipulated in vignette)

*p < .05

## Study 3

### Method

In study 3, we ascertained the test-rest reliability (stability) and sensitivity to change (responsiveness) of the new measures in an online experimental study with a subsample of the respondents from study 2. Test-retest reliability refers to the extent to which test scores are consistent over time [24]. Sensitivity to change refers to the extent to which test scores detect a change in circumstances [24]. Sensitivity to change, or responsiveness, is one of Glasgow and Riley’s required criterion for pragmatic measures [11].

#### Design, participants, and procedures

Half of participants in study 2 were randomly assigned to receive the same vignette they received earlier (test-retest reliability); the other half were randomly assigned to receive the exact opposite vignette they received earlier (sensitivity to change). For example, a participant who earlier received a vignette in which the factors were high-high-low would receive a vignette in which the factors are low-low-high. We stratified the random assignment across vignettes to ensure balance in the conditions for the test-retest and sensitivity analysis.

Of the 326 counselors who participated in study 2, 296 provided email addresses for a follow-up survey. These individuals received an email invitation to participate in study 3, with non-respondents receiving up to three reminder emails spaced a week apart. One-hundred ninety-two participants (65%) responded to the follow-up survey. Twenty-six percent identified as male, 73% as female. Two participants identified as non-binary gender. Ninety-four percent identified as Caucasian, 2% as African American, 2% as Asian, and 2% as (‘Other’). Five percent identified as Hispanic, Latino, or Spanish origin. Seventy-four percent indicated they worked in a private practice setting. Fifty-five percent reported that they had implemented an EBP or led the implementation of an EBP in the past 5 years. Of the 192 study participants responding to the follow-up survey, 95 generated data for the test-retest reliability assessment and 97 generated data for the sensitivity to change assessment.

We used the same data collection procedures we used in study 2. Study 3 began 3 weeks after study 2 concluded. Median response time between surveys was 7 weeks.

#### Measures

We used the same measures as in study 2. That is, we used the short (4-item) version of the AIM, IAM, and FIM with 5-point ordinal response options (Additional file 3).

#### Data analysis

We assessed inter-item consistency by computing Cronbach alphas for the four-item scales. We assessed test-retest reliability by calculating Pearson correlation coefficients corrected for attenuation due to measurement error. Disattenuation involved dividing the Pearson correlation coefficient by the square root of the product of the Cronbach alphas for the scales [25]. Disattenuated scales demonstrating correlations greater than 0.70 were considered test-retest reliable. We assessed sensitivity to change using linear regression models to predict the difference score (or change in the implementation outcome measure) based on whether the vignette assignment order was low-high, high-low, or high-high; the assignment order low-low served as the reference group.

### Results

As previously noted, Cronbach alphas for the 4-item scales from the structural validity survey (study 2) were 0.85 for acceptability, 0.91 for appropriateness, and 0.89 for feasibility. The Cronbach alphas for the scales from the test-retest reliability survey were 0.83 for acceptability, 0.87 for appropriateness, and 0.88 for feasibility. Two observations were removed as bivariate outliers for the appropriateness scales, dropping the *N* to 93 for the test-retest reliability assessment. The Pearson correlation coefficients corrected for attenuation due to measurement error were 0.80 for acceptability, 0.73 for appropriateness, and 0.88 for feasibility. All three correlations exceeded 0.70; hence, the three measures demonstrated acceptable test-retest reliability.

Regression analysis indicated that vignette assignment order explained 41, 42, and 46% of the variance in change in the acceptability, appropriateness, and feasibility measures, respectively (see Table 3). The regression coefficients for the assignment order low-high and high-low were statistically significant and signed in the expected direction for each implementation outcome. These results indicate that each measure was sensitive to change in both directions, from low to high and high to low, as study participants responded to the information manipulated in the vignettes.

**Table 3:** Regression analysis of sensitivity to change, *N* = 97

	Acceptability			Appropriateness			Feasibility		
	beta	SE	*p* value	beta	SE	*p* value	beta	SE	*p* value
Intercept	− 0.06	0.10	0.5732	0.10	0.12	0.4192	0.09	0.13	0.4816
Vignette 1 low on construct Vignette 2 high on construct	0.76	0.15	< .0001	0.68	0.17	< .0001	0.92	0.18	< .0001
Vignette 1 high on construct Vignette 2 low on construct	− 0.90	0.14	< .0001	− 1.18	0.17	< .0001	− 1.26	0.18	< .0001
Vignette 1 high on construct Vignette 2 high on construct	0.02	0.15	0.9082	− 0.20	0.16	0.2093	− 0.10	0.17	0.5744
Adjusted *R* ^2^	0.41			0.42			0.46		

## Discussion

This study sought to advance implementation science by systematically developing valid, reliable, and pragmatic measures of three key implementation outcomes: acceptability (Acceptability of Intervention Measure (AIM)), appropriateness (Intervention Appropriateness Measure (IAM)), and feasibility (Feasibility of Intervention Measure (FIM)). Substantive and discriminant content validity assessment involving implementation researchers and implementation-experienced mental health professionals indicated that most of the items that we generated reflected the conceptual content of these three implementation outcomes. Exploratory and confirmatory factor analyses produced brief 5-item scales with acceptable model fit and high reliability. Although the scales were highly correlated, nested confirmatory factor analysis models provided evidence that the three implementation outcomes are best represented from an empirical perspective as distinguishable constructs, just as Proctor and colleagues suggest [1].

Scale refinement through construct-specific confirmatory factor analysis of data obtained from a vignette study involving practicing mental health counselors resulted in trimmed 4-item scales. Nested confirmatory factor analysis models provided evidence of structural validity, with the three-factor model demonstrating acceptable model fit and high-scale reliability. Analysis of variance provided evidence of known-groups validity, with medium- to large-size main effects of each manipulation on the relevant scale score. Although the design error precluded a full exploration of discriminant validity, the analysis of variance indicated that the newly developed measures of acceptability, appropriateness, and feasibility can differentiate groups with known differences in the levels of these implementation outcomes.

Finally, test-retest reliability and sensitivity to change were demonstrated when a subsample of mental health counselors participating in the vignette study randomly received either the same vignette or the opposite vignettes and re-rated the implementation outcomes. These measurement properties are important to researchers and other stakeholders (e.g., intermediaries, policymakers, practice leaders) yet are rarely assessed. Importantly, regression analysis indicated that the implementation outcome measures were sensitive to change in both directions: high to low and low to high. This makes the measures useful for assessing the impact of planned strategies or unexpected events on practitioners’ perceptions of acceptability, appropriateness, and feasibility.

### Contributions to implementation science and practice

This study contributes to the literature by developing valid and reliable measures of important implementation outcomes [1]. The field of implementation science has been deemed a Tower of Babel given the lack of conceptual clarity and consistency in its terminology [26], and concerns about the state of measurement in the field have been well documented [3, 27, 28]. These concerns extend to the measurement of implementation outcomes, as, despite their centrality to understanding the extent to which implementation is successful, valid and reliable measures are lacking [2]. This study fills that gap by developing valid and reliable measures of three implementation outcomes that are salient to a wide range of implementation studies as well as a wide range of pilot, efficacy, and effectiveness studies. Indeed, with an increasing focus on designing for dissemination and implementation [29], and some going as far as to say that all effectiveness studies be Hybrid I effectiveness-implementation studies [30, 31], measuring constructs such as acceptability, appropriateness, and feasibility will be a ubiquitous need. These outcomes are relevant to assessing stakeholders’ perceptions of clinical and public health interventions, as well as assessing perceptions of implementation strategies, which are often complex interventions in and of themselves [1, 32, 33]. Assessing these outcomes early in the research process may ensure that interventions and implementation strategies are optimized and fit with end-users’ preferences.

In addition to the need for valid and reliable measures of implementation-related constructs, there is a need for pragmatic measures [11]. Implementation stakeholders are unlikely to use measures unless they possess these qualities, which may include broad domains such as being (1) useful in informing decision-making, (2) compatible with the settings in which they are employed, (3) easy to use, and (4) acceptable [34]. Pragmatic measures are particularly important for low-resource settings [35]. There are several examples of recent efforts to develop pragmatic measures for implementation constructs such as organizational readiness for change [36], implementation leadership [37], and implementation climate [38, 39]. We sought to ensure that our measures were pragmatic, and we believe that we have accomplished that in three ways. First, we sought to develop measures that were brief. We began by developing 12 or fewer items for each construct, and our psychometric testing resulted in final measures with only four items per construct. Second, we made each item as general as possible by not specifying a specific context or clinical problem within the items. For example, the appropriateness items did not specify a purpose (e.g., “for treating depression”), person (e.g., “for my patients”), or situation (e.g., “for my organization”); those wishing to use these measures could add such referents to explore specific aspects of appropriateness (i.e., social or technical fit). Third, we purposefully made the measures “open access” to ensure that the scales are freely available to all who might wish to use them. Our hope is that developing a measure that is free, brief, easy to use and not context- or treatment-specific will increase the chances of its use broadly in implementation research and practice. One of the unfortunate realities in implementation science is that, to date, the majority of current measures are developed for the purpose of a single study (usually with minimal conceptual clarity and psychometric testing) and then never used again. This state of affairs precludes our ability to develop generalizable knowledge in implementation science and, more specifically, knowledge about how current measures perform across a wide range of contexts. Of course, whether these measures ultimately demonstrate predictive validity within the field of mental health and other clinical settings is yet to be determined.

Finally, we have laid out a systematic process for measure development and testing that we believe is both replicable and feasible. In doing so, we stress the importance of clearly defining constructs and engaging in domain delineation processes that ensure that constructs are sufficiently differentiated from similar constructs. We also stress careful psychometric testing, and lay out a process for establishing substantive validity, discriminate validity, structural validity, discriminant validity, known-groups validity, test-retest reliability, and sensitivity to change. The measurement development and testing process took 15 months, suggesting that the process could be completed within the period of a grant-funded implementation study. We encourage other teams to replicate this methodology and suggest further refinements that may enhance the efficiency and effectiveness of this process.

### Limitations

Though there were a number of strengths in the current study, there were also limitations. First, correlations among the three factors of acceptability, appropriateness, and feasibility were at times fairly high and discriminant validity was not fully tested due to a survey design error. Future research would benefit from further explorations of the discriminant validity of these constructs.

Replication is also needed. Testing the measures with samples of providers that have different backgrounds and characteristics than the samples included here would yield important information about generalizability (e.g., structural invariance). Likewise, testing the measures with different methods or materials would also be useful.

### Future directions

We plan to administer prospectively our newly developed measures to a large sample of providers faced with the decision to adopt, or not adopt, an EBP. We will then evaluate whether their perceptions of the acceptability, appropriateness, and feasibility of the EBP predict their adoption of the EBP. If the predictive validity of our measures is established, researchers and practitioners would have a brief tool for assessing early on if staff are likely to adopt an EBP or if more work needs to be done to increase the EBP’s acceptability, appropriateness, or feasibility.

Glasgow and Riley [11] argue that measures need to be pragmatic if they are to be useful (and used) outside the context of research. Pragmatic features of measures include sensitivity to change, brevity, psychometric strength, actionability, and relevance to stakeholders. While their list of pragmatic features is helpful, it was not developed with stakeholder input and therefore might not reflect what stakeholders view as important. To address this limitation, we are working with stakeholders to define and operationalize pragmatic features of measures, with the goal of developing rating criteria that can be used to assess the pragmatic properties of measures, much like the psychometric properties of measures are assessed [4]. In future work, we will apply these pragmatic rating criteria to the three new measures of implementation outcomes developed here.

## Conclusions

In sum, we developed three new measures (the Acceptability of Implementation Measure, Implementation Appropriateness Measure, and Feasibility of Intervention Measure) that are considered to be important implementation outcomes in their own right as well as leading indicators of other implementation outcomes, such as adoption. Our development procedures resulted in 12 items (four for each construct) that are both valid and reliable measures of these implementation outcomes. Predictive validity will be assessed in a forthcoming prospective follow-up study. We will also subject these measures to a formal evaluation of pragmatic properties, testing features beyond their brief nature and sensitivity to change. These measures have great potential for widespread use across implementation studies regardless of intervention focus, target disease/problem, and setting because of their general wording, boosting their ability to generate cumulative knowledge. Moreover, the measure development process employed in this set of studies presents a replicable and relatively efficient method.

## Additional files

Additional file 1: Exploratory factor analysis (EFA) using promax rotation in *Mplus* 7. This file includes the EFA data from the web-based survey results in which 31 items were assigned to three constructs (acceptability, appropriateness, and feasibility). (DOCX 28 kb)

Additional file 2: Scale reliabilities and factor loadings from single-factor confirmatory factor analysis of structural validity vignette data, *N* = 326. This file includes factor loadings in the one-factor CFAs. (DOCX 16 kb)

Additional file 3: Final version of the Acceptability of Intervention Measure (AIM), Intervention Appropriateness Measure (IAM), and Feasibility of Intervention Measure (FIM). (DOCX 16 kb)

## Abbreviations

AIM: Acceptability of Intervention Measure
AMHCA: American Mental Health Counselors Association
ANOVA: Analysis of variance
CFA: Confirmatory factor analysis
CFI: Comparative fit index
EBP: Evidence-based practice
EFA: Exploratory factor analysis
FIM: Feasibility of Intervention Measure
IAM: Intervention Appropriateness Measure
ICC: Intraclass correlation coefficients
MBC: Measurement-based care
RMSEA: Root mean square error of approximation
