# Supplementary material for: Psychometric assessment of three newly developed implementation outcome measures
Source: Implement Sci. 2017 Aug 29;12:108. doi: 10.1186/s13012-017-0635-3 (PMC5576104; doi:10.1186/s13012-017-0635-3)
Supplement: Supplementary file 1 — Exploratory factor analysis (EFA) using promax rotation in Mplus 7. This file includes the EFA data from the web-based survey results in which 31 items were assigned to three constructs (acceptability, appropriateness, and feasibility). (DOCX 28 kb) [file 13012_2017_635_MOESM1_ESM.docx]

Additional File 1. Exploratory factor analysis (EFA) using promax rotation in M*plus* 7

|  | **ACCEPTABLE** | | | | |  | **APPROPRIATE** | | | | |  | **FEASIBLE** | | | | |
| --- | --- | --- | --- | --- | --- | --- | --- | --- | --- | --- | --- | --- | --- | --- | --- | --- | --- |
|  | Factor 1 | Factor 2 | Factor 3 | Factor 4 | Factor 5 |  | Factor 1 | Factor 2 | Factor 3 | Factor 4 | Factor 5 |  | Factor 1 | Factor 2 | Factor 3 | Factor 4 | Factor 5 |
| This EBP seems fine. | 0.42 | 0.18 | -0.04 | **0.78** | 0.13 |  | 0.20 | 0.36 | 0.29 | -0.32 | 0.02 |  | -0.04 | 0.00 | **0.88** | 0.16 | -0.05 |
| This EBP seems good enough. | **0.52** | -0.01 | -0.01 | **0.47** | -0.10 |  | 0.23 | **0.58** | -0.08 | 0.16 | 0.14 |  | -0.13 | 0.17 | **0.83** | 0.23 | 0.10 |
| This EBP will do. | **0.63** | 0.08 | 0.06 | 0.27 | -0.15 |  | 0.21 | 0.37 | 0.28 | **0.55** | 0.26 |  | -0.11 | 0.29 | **0.85** | 0.11 | -0.05 |
| This EBP meets my approval. | **0.69** | 0.20 | -0.04 | 0.16 | 0.30 |  | 0.05 | -0.08 | 0.82 | -0.21 | 0.34 |  | -0.02 | -0.20 | **0.90** | -0.19 | 0.31 |
| This EBP meets my needs. | **0.53** | **0.57** | 0.14 | 0.16 | 0.08 |  | 0.01 | 0.05 | **0.38** | **0.35** | 0.12 |  | -0.22 | -0.03 | **0.83** | -0.04 | 0.12 |
| This EBP seems okay. | **0.53** | 0.06 | -0.07 | **0.65** | 0.08 |  | 0.28 | **0.46** | **0.67** | 0.09 | 0.01 |  | -0.04 | 0.26 | **0.88** | 0.07 | -0.04 |
| This EBP is satisfactory. | **0.54** | 0.12 | -0.16 | **0.56** | 0.11 |  | 0.03 | 0.45 | **0.67** | 0.14 | 0.16 |  | 0.00 | 0.15 | **0.91** | 0.26 | -0.02 |
| I have no objection to this EBP. | **0.68** | 0.13 | -0.13 | 0.24 | 0.42 |  | 0.08 | -0.15 | **0.71** | 0.01 | 0.48 |  | 0.00 | -0.26 | **0.81** | -0.03 | 0.47 |
| This EBP is pretty good. | **0.59** | 0.18 | -0.10 | **0.58** | 0.04 |  | 0.23 | **0.46** | **0.45** | 0.04 | -0.01 |  | 0.02 | 0.20 | 0.82 | 0.24 | -0.02 |
| This EBP is appealing. | **0.66** | 0.13 | -0.02 | 0.24 | **0.48** |  | -0.09 | 0.05 | 0.73 | 0.21 | 0.05 |  | -0.39 | -0.12 | 0.83 | -0.18 | 0.33 |
| I like this EBP. | **0.74** | 0.25 | 0.04 | 0.19 | 0.48 |  | -0.31 | -0.27 | **0.87** | 0.02 | 0.27 |  | -0.33 | -0.27 | **0.88** | -0.04 | 0.32 |
| I welcome use of this EBP | **0.71** | 0.21 | 0.05 | 0.15 | **0.53** |  | -0.11 | -0.17 | **0.85** | -0.06 | 0.20 |  | -0.15 | -0.23 | **0.90** | -0.14 | 0.25 |
| This EBP seems right. | 0.16 | -0.23 | **0.61** | 0.35 | 0.20 |  | **0.43** | 0.15 | 0.14 | 0.06 | **0.34** |  | -0.12 | -0.11 | **0.89** | -0.11 | 0.31 |
| This EBP seems fitting. | 0.14 | 0.28 | **0.85** | 0.14 | 0.16 |  | **0.79** | 0.22 | -0.15 | 0.42 | -0.06 |  | -0.02 | 0.17 | **0.83** | 0.19 | 0.33 |
| This EBP seems suitable. | 0.07 | 0.19 | **0.79** | 0.19 | 0.14 |  | **0.73** | 0.35 | -0.05 | 0.37 | -0.01 |  | -0.13 | 0.23 | **0.77** | 0.23 | 0.29 |
| This EBP seems reasonable. | 0.24 | -0.37 | **0.52** | 0.30 | 0.20 |  | 0.23 | 0.04 | 0.32 | -0.01 | 0.27 |  | **0.34** | -0.07 | 0.51 | -0.03 | **0.14** |
| This EBP seems applicable. | -0.14 | -0.09 | **0.77** | -0.12 | 0.21 |  | **0.80** | 0.08 | -0.24 | 0.07 | 0.06 |  | -0.14 | -0.01 | **0.78** | -0.13 | 0.35 |
| This EBP seems right on the button. | 0.33 | -0.16 | **0.53** | 0.07 | 0.23 |  | **0.50** | -0.02 | 0.24 | 0.31 | 0.09 |  | 0.00 | -0.31 | **0.86** | -0.09 | 0.14 |
| This EBP seems proper. | 0.22 | 0.00 | **0.52** | **0.68** | -0.04 |  | **0.52** | **0.54** | 0.09 | 0.09 | -0.07 |  | -0.07 | 0.16 | **0.98** | 0.32 | 0.06 |
| This EBP seems apt. | 0.04 | 0.15 | **0.57** | **0.47** | 0.06 |  | **0.52** | **0.49** | -0.04 | 0.33 | 0.05 |  | 0.21 | 0.14 | 0.77 | **0.49** | 0.31 |
| This EBP seems like a good match. | 0.04 | 0.34 | **0.70** | -0.05 | **0.58** |  | **0.76** | 0.11 | -0.13 | 0.30 | 0.01 |  | -0.05 | 0.06 | **0.85** | -0.07 | 0.44 |
| This EBP seems well aligned. | 0.10 | 0.18 | **0.74** | 0.13 | 0.18 |  | **0.68** | 0.20 | -0.02 | 0.33 | -0.13 |  | -0.15 | 0.11 | **0.79** | 0.06 | 0.22 |
| This EBP seems practical. | -0.20 | -0.02 | **0.65** | 0.21 | **0.61** |  | 0.21 | 0.11 | 0.46 | -0.03 | **0.63** |  | **0.65** | -0.12 | 0.08 | -0.09 | -0.14 |
| This EBP seems realistic. | 0.00 | -0.04 | **0.66** | 0.28 | **0.67** |  | 0.10 | 0.12 | **0.67** | 0.04 | 0.65 |  | **0.62** | -0.04 | 0.03 | -0.24 | -0.12 |
| This EBP seems workable. | -0.20 | -0.23 | **0.72** | 0.23 | **0.63** |  | -0.05 | -0.15 | **0.62** | 0.39 | **0.63** |  | **0.69** | 0.22 | 0.12 | -0.12 | 0.06 |
| This EBP seems implementable. | -0.19 | 0.22 | **0.85** | 0.29 | 0.30 |  | -0.11 | 0.23 | **0.74** | 0.37 | 0.35 |  | **0.79** | 0.34 | -0.05 | 0.17 | -0.08 |
| This EBP seems possible. | -0.16 | -0.05 | **0.91** | 0.05 | 0.50 |  | -0.19 | 0.01 | **0.89** | -0.13 | 0.49 |  | **0.87** | 0.05 | -0.25 | -0.01 | -0.04 |
| This EBP seems viable. | 0.06 | -0.39 | **0.79** | 0.12 | 0.06 |  | 0.05 | -0.05 | **0.68** | -0.20 | 0.23 |  | **0.59** | 0.22 | -0.08 | 0.09 | 0.35 |
| This EBP seems doable. | -0.17 | 0.25 | **0.91** | 0.19 | 0.33 |  | -0.14 | 0.30 | **0.87** | 0.26 | 0.40 |  | **0.91** | 0.26 | -0.20 | 0.15 | -0.27 |
| This EBP seems easy to use. | -0.24 | -0.04 | **0.78** | 0.29 | **0.65** |  | -0.23 | -0.12 | **0.88** | 0.01 | 0.52 |  | **0.90** | 0.06 | -0.15 | 0.01 | -0.07 |
| This EBP seems challenging. | -0.14 | 0.15 | **0.54** | 0.28 | 0.57 |  | -0.03 | 0.11 | **0.92** | -0.01 | 0.17 |  | **0.76** | -0.10 | -0.17 | 0.25 | -0.36 |
| Note: **Bold** text indicates on which factor the item is loaded. | | | | | | | | | | | | | | | | | |
