# Supplementary material for: Psychometric assessment of three newly developed implementation outcome measures
Source: Implement Sci. 2017 Aug 29;12:108. doi: 10.1186/s13012-017-0635-3 (PMC5576104; doi:10.1186/s13012-017-0635-3)
Supplement: Supplementary file 2 — Scale reliabilities and factor loadings from single-factor confirmatory factor analysis of structural validity vignette data, N = 326. This file includes factor loadings in the one-factor CFAs. (DOCX 16 kb) [file 13012_2017_635_MOESM2_ESM.docx]

Additional File 2. Scale Reliabilities and Factor Loadings from Single-factor Confirmatory Factor Analysis of Structural Validity Vignette Data, *N* = 326

|  | | Cronbach’s ɑ  5-item SCALE | 5 ITEM FACTOR LOADINGS | Cronbach’s ɑ  4-item SCALE | 4 ITEM FACTOR LOADINGS |
| --- | --- | --- | --- | --- | --- |
| **Acceptable** | | .84 |  | .85 |  |
|  | MBC meets her approval. |  | .68 |  | .77 |
|  | She has no objection to this MBC. |  | .50 |  | . |
|  | MBC is appealing to her. |  | .77 |  | .83 |
|  | She likes this MBC. |  | .85 |  | .74 |
|  | She welcomes MBC. |  | .80 |  | .77 |
| **Appropriate** | | .92 |  | .91 |  |
|  | MBC seems fitting. |  | .86 |  | .86 |
|  | MBC seems suitable. |  | .85 |  | .87 |
|  | MBC seems applicable. |  | .79 |  | .81 |
|  | MBC seems like a good match. |  | .85 |  | .83 |
|  | *MBC* seems well aligned. *(5 item scale only)* |  | .81 |  | . |
| **Feasible** | | .92 |  | .89 |  |
|  | MBC seems implementable. |  | .83 |  | .83 |
|  | MBC seems possible. |  | .77 |  | .78 |
|  | MBC seems doable. |  | .91 |  | .90 |
|  | MBC seems easy to use. |  | .74 |  | .75 |
|  | MBC seems workable.  *(5 item scale only)* |  | .91 |  | . |
